# Current Progress and Perspective: Clinical Imaging of Islet Transplantation

**DOI:** 10.3390/life10090213

**Published:** 2020-09-19

**Authors:** Taylor Marie Richards, Aixia Sun, Hasaan Hayat, Neil Robertson, Zhaoda Zhang, Jinda Fan, Ping Wang

**Affiliations:** 1 Precision Health Program, Michigan State University, East Lansing, MI 48823, USA; rich1035@msu.edu (T.M.R.); sunaixia@msu.edu (A.S.); hayathas@msu.edu (H.H.); robe1104@msu.edu (N.R.); 2 Department of Radiology, College of Human Medicine, Michigan State University, East Lansing, MI 48824, USA; 3 Lyman Briggs College, Michigan State University, East Lansing, MI 48002, USA; 4 Athinoula A. Martinos Center for Biomedical Imaging, Department of Radiology, Massachusetts General Hospital and Harvard Medical School, Charlestown, MA 02129, USA; 5 Institute for Quantitative Health Science and Engineering, Michigan State University, East Lansing, MI 48824, USA; 6 Department of Chemistry, College of Natural Science, Michigan State University, East Lansing, MI 48824, USA

**Keywords:** type 1 diabetes (T1D), islet transplantation, beta cell mass (BCM), magnetic resonance Imaging (MRI), positron emission tomography (PET), single photon emission computed tomography (SPECT)

## Abstract

Islet transplantation has great potential as a cure for type 1 diabetes. At present; the lack of a clinically validated non-invasive imaging method to track islet grafts limits the success of this treatment. Some major clinical imaging modalities and various molecular probes, which have been studied for non-invasive monitoring of transplanted islets, could potentially fulfill the goal of understanding pathophysiology of the functional status and viability of the islet grafts. In this current review, we summarize the recent clinical studies of a variety of imaging modalities and molecular probes for non-invasive imaging of transplanted beta cell mass. This review also includes discussions on in vivo detection of endogenous beta cell mass using clinical imaging modalities and various molecular probes, which will be useful for longitudinally detecting the status of islet transplantation in Type 1 diabetic patients. For the conclusion and perspectives, we highlight the applications of multimodality and novel imaging methods in islet transplantation.

## 1. Introduction

The selective and progressive destruction of beta cells within the islets of Langerhans in the pancreas by autoimmunity leads to type 1 diabetes (T1D) [1]. Without the production of a sufficient amount of insulin, the body is unable to effectively control blood glucose levels. T1D affects 387 million people worldwide of different ages, ethnic groups and genders [2]. Currently T1D is treated using an exogenous insulin replacement. While administration of insulin has been proven to be able to control hyperglycemia and delay progression of some complications [3], it does not fully restore glucose homeostasis and cure T1D [4], leading to a variety of disease-associated complications. Many research projects and clinical trials have been focused on the possible cures for T1D, including pancreatic islet transplantation. Islet transplantation has a lifesaving potential for curing T1D patients [5]. For a successful T1D cell therapy, the islets must first implant and then persist for years. However, even with the success of the Edmonton immunosuppressive protocol, only 20% of patients remain insulin-independent for 3 years after islet transplantation [6]. To understand why some transplants fail and how to improve the treatment, we need to have a method that enables us to track the function and viability of the transplanted islets over time so we can detect rejection and loss when it occurs. Unfortunately, it is currently only possible to estimate islet mass and the fate of transplantation indirectly, through measurement of circulating C-peptide, insulin and glucose levels. These assays could not provide quantitative assessment of surviving islets, because the effect of insulin secretion of islets is non-linear, and having even a small number of active islets can secrete insulin and lead to a dramatic drop in blood glucose level. Thus, the blood glucose level is a lagging indicator of the success of the islet transplantation. The development of a direct quantitative measure of surviving islets would improve our ability to perform research on new approaches to hurdle immune rejection and allow the detection and intervention of transplantation loss in human islet transplantation. For example, if we were able to detect an increase in the loss rate of a patient’s islet transplantation, we could change their immune-suppressing drug regimen. The most promising approaches for directly measuring islet health in vivo have been based on medical imaging tools such as positron emission tomography (PET), single photon emission computed tomography (SPECT) and magnetic resonance imaging (MRI) [7].

The discovery of a reliable imaging technique that permits reliable, sensitive and accurate imaging of the beta cells would not only help realize new treatment of curing T1D with islet transplantation, but also allow further studying into the progression and the epidemiology of diabetes, as well as developing new theranostic strategies. Although transplanted islet cells are not located at pancreas, they share similarity with endogenous pancreatic beta cells. Thus, we review the clinical studies of a variety of imaging modalities and molecular probes for detecting transplanted and endogenous beta cell mass.

## 2. Nuclear Imaging

Nuclear imaging such as PET and SPECT are important clinical diagnostic and research modalities, which offer high sensitivity and possible quantification. As non-invasive medical and molecular imaging techniques, PET and SPECT have offered the capability of assessing biological processes at the cellular and molecular levels in vivo, which offer the possibility to visualize and analyze the target molecule change under physiological and pathophysiological conditions.

**Figure 1:**
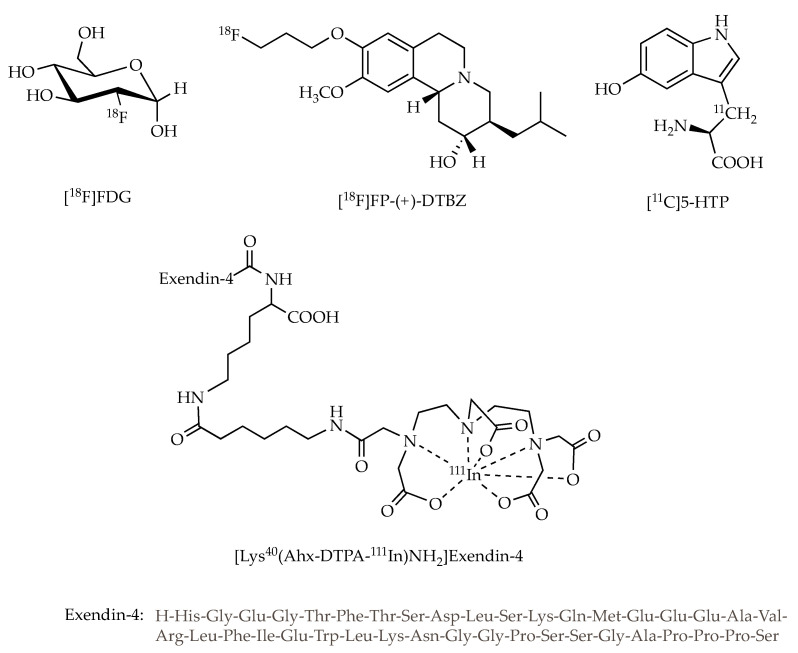
Schematic structures of [^18^F]FDG, [^18^F]FP-(+)-DTBZ, [^11^C]5-HTP and [Lys^40^(Ahx-DTPA-^111^In)NH_2_]Exendin-4.

Despite the great prosperity of information that such modalities can deliver, the potential of nuclear imaging depends strongly on the availability of selective and effective radiotracers. In addition, the sensitivity of nuclear imaging depends not only on the selectivity of tracers to targets but also on the specific radioactivity of tracers. Because the beta cell target mass is extremely small and the molecular target level associated with beta cell is very low, the tracers require high specific radioactivity to be effective to specifically image beta cells. Thus, extensive research effort has been directed towards the development of highly beta-cell selective radioligands with high specific radioactivity for probing beta cells by targeting cell-specific receptors, antigens and metabolites [8,9,10,11], in which many radiotracers have been reported and several of which, such as [^18^F]fluorodeoxyglucose ([^18^F]FDG), [^18^F]fluoropropyl-dihydrotetrabenazine ([^18^F]FP-(+)-DTBZ) and [^11^C]5-hydroxy-tryptophan ([^11^C]5-HTP) for PET, and [Lys^40^(Ahx-DTPA-^111^In)NH2]exendin-4 (Ahx is aminohexanoic acid and DTPA is diethylenetriaminepentaacetic acid) for SPECT (Figure 1), have been advanced to human clinical trials. To date, the molecular probes used to image in vivo pancreatic beta cells mentioned above have focused on two different approaches: ex vivo labelling and in vivo targeting. Initial reports to track transplanted islets employed the direct cell-radiolabeling. In recent years, selective tracers targeting the biomarkers on the surface of islet beta cells or the biosynthesis involved in beta cells have been developed, with which the mass of endogenous beta cells in the pancreas and transplanted islets in the liver as well as in the muscle were quantitatively determined using PET or SPECT imaging.

### 2.1. Positron Emission Tomography (PET) Imaging of Transplanted Islet with [^18^F]Fluorodeoxyglucose ([^18^F]FDG)

[^18^F]FDG measures the glucose metabolism of cells and tissues. Ex vivo labelling of islets is a practical method for real-time evaluating engraftment and distribution during the transplantation procedure, in which [^18^F]FDG had been studied in the clinical islet transplantation.

#### [^18^F]fluorodeoxyglucose ([^18^F]FDG)

[^18^F]FDG is the most common PET radiotracer used both clinically and preclinically for different diseases such as cancers and Alzheimer’s disease. As an F-18 labelled glucose analog, [^18^F]FDG is a substrate for glucose transporter (GLUT) and for phosphorylation by hexokinase, in which the resultant product 2-[^18^F]FDG-6-phosphate does not proceed further in the glycolytic pathway and is trapped in the living cell as an indicator of the glycolysis rate of cells [12]. Since [^18^F]FDG is widely available, it is an attractive tracer for ex vivo labeling of islets to image their distribution and kinetics during and after clinical transplantation.

In 2009, Eriksson et al. reported that their dynamic PET/CT study using [^18^F]FDG-labeled islets permitted qualitative and quantitative analysis for 60 min during islet transplantation in five T1D patients receiving six transplants [13]. In this study, the islets were incubated with [^18^F]FDG for 60 min at 37 °C, and then 23% of the labeled islets were mixed with unlabeled islets (77%) just prior to intraportal transplantation. The results show that the peak radioactivity in the liver was found at 19 min after the start of islet infusion and maximum uptake of radioactivity varied but the time–activity curves during transplantation were similar for the four main liver segments, indicating that islet delivery to the different segments was constant during the transplantation procedure, although the distribution of transplanted islets was heterogeneous with wide variations in location and concentration in the liver.

It was found that the mean maximum uptake of labeled islets in the liver was 63% of the infused radioactivity. Assumed that 16% of the radioactivity may have left the graft meantime, therefore, only 75% of the radioactivity confined within the islets (63% out of remaining 84%) stayed in the liver, indicating that islets are lost during the transplantation procedure. They also found that distribution in the liver was heterogeneous with wide variations in location and concentration. Some hot spots of radioactivity were found within each liver segment, most likely representing islets trapped in sinusoids or in clots in the portal branches. Islets found in areas with concentrations of >400 islet equivalents (IEQ)/cc liver tissue varied between 1% and 32% of the graft in different subjects. A better correlation to clinical outcome was achieved for this group of five patients when considering islets in volumes of >300 (IEQ)/cc as nonfunctional. Therefore, this study revealed that islets entrapped in hot spots with densities above a specified cut-off value (e.g., >300 (IEQ)/cc) and islets lose during the transplantation procedure were two reasons why islet graft function was estimated to be as low as a fifth of that of a healthy person, even in patients becoming insulin independent after islet transplantation. It is indicated that the data processing method for this PET imaging should be optimized to correctly estimate the number/density of the functional islets. Furthermore, the sensitivity of PET imaging may limit the detection of single islets, leading to underestimation of infused islets.

The results also showed that no detectable side effects attributed to the PET/computed tomography (CT) procedure were discovered and clinical outcome in all patients in this study was comparable to that previously reported and observed from non-radiolabeled islet transplantations, indicating that the [^18^F]FDG labeling procedure did not harm the islets.

The main advantage of this approach is that [^18^F]FDG is readily available and provides real-time quantitative and qualitative analysis of islet kinetics and distribution during the first hour after transplantation. However, the results should be interpreted with caution due to the limited number of patients in this study. On the other hand, the half-life of ^18^F (109.8 min) and retention of [^18^F]FDG in islets (196 min) limits its use for 1 h. Although a PET radiotracer labeled with isotope of longer half-life may be able to monitor the long-term islet survival, which will depend not only on how long the tracer can be associated with islets, but also on whether the higher radiation dosimetry associated with longer half-life radioisotopes causes damage to the islets and health. [^18^F]FDG PET imaging is based on cell metabolic rates, thus it lacks specificity to islets in comparison to surrounding tissues. This also limits the application of [^18^F]FDG PET imaging of beta cell mass (BCM) in longitudinal studies after the transplantation. Thus beta cell selective probes other than [^18^F]FDG are needed to enhance the retention of the tracer in islets.

### 2.2. PET Imaging of Beta Cell Associated Targets

In vivo targeting is more versatile and clinically applicable method, as transplanted patients can be investigated longitudinally with suitable tracers, in which some established PET or SPECT neurotransmission or neuroendocrine tumor imaging agents have been repurposed for imaging of beta cells. These radioligands have been developed to target vesicular monoamine transporter 2 (VMAT2), serotonin biosynthesis and glucagon-like peptide-1 receptor (GLP-1R).

#### 2.2.1. [^18^F]fluoropropyl-dihydrotetrabenazine ([^18^F]FP-(+)-DTBZ)

[^18^F]FP-(+)-DTBZ is an analogue of DTBZ with improved affinity and pharmacological profile, serving as PET radiotracers for imaging of vesicular monoamine transporter 2 (VMAT2) [14,15]. As a membrane-spanning protein, VMAT2 is mainly associated with the dopaminergic system and has its effect by transporting biogenic monoamines into secretory vesicles. Since VMAT2 is found to be co-localized with insulin in beta cells, it has been studied as a biomarker for BCM imaging. 

In 2013, a clinical PET study for imaging VMAT2 with [^18^F]FP-(+)-DTBZ was carried out to evaluate BCM in 4 healthy control subjects and 3 patients with T1D mellitus [16]. In the study, the radioactivity in the pancreas was normalized by injected dose and body weight. The results showed that standardized uptake value (SUV) was 37.7% lower in the pancreas of T1D mellitus patients than in control subjects. The results also suggested that the 32% reduction in pancreas volume together with the 40% decrease in VMAT2 binding potential (BP_ND_ , the tracer-specific binding per unit volume of tissue) gave a calculated 59% loss in total [^18^F]FP-(+)-DTBZ uptake in the pancreas of C peptide–negative T1D mellitus patients, compared with the corresponding healthy individuals.

To image the pancreas with PET is likely limited by the partial-volume effect, which may lose apparent activity in small objects or regions and cause an underestimation of the measured radioactivity concentration. To minimize partial volume effects in this study, the regions of interest (ROI) were drawn with caution to ensure the areas of the edge of the pancreas that could be susceptible to signal spill-out were excluded. In addition, the contribution from non-specific binding was corrected by use of the kidney as the reference region. They used the volume of distribution (V_T_) in the kidney as a measure of non-specific uptake to determine the specific binding index (BP_ND_) in the pancreas. The binding parameters V_T_ and BP_ND_ of the tracer were found to correlate with insulin secretion capacity as determined from arginine stimulus tests, displaying the relationship between [^18^F]FP-(+)-DTBZ binding in the pancreas and beta cell function.

In 2016, a new clinical trial was carried out to further study the PET imaging of the pancreatic [^18^F]FP-(+)-DTBZ uptake and binding to assess VMAT2 as a biomarker of BCM in a relatively larger cohort, which included 14 healthy controls, 8 patients with T1D and 3 patients with Type 2 Diabetes (T2D) [17]. In the trial, the PET signal that depends on VMAT2 binding was decreased in patients with longstanding T1D when compared to healthy controls. More specifically, the functional binding capacity (BP_ND_ × volume) in T1D patients was reduced 63% compared to healthy controls, which is similar to that (59%) reported by Normandin et al. [16]. The results indicated that the pancreatic VMAT2 binding was significantly decreased in patients with T1D compared to groups of healthy controls, which suggests that PET imaging could potentially detect beta cell loss by using [^18^F]FP-(+)-DTBZ tracer [17]. On the other hand, this was the first time that [^18^F]FP-(+)-DTBZ was tested on T2D patients. The results indicated that there was no significant difference between T2D patients and the healthy control group for VMAT2 binding. Additionally, this study also checked the reproducibility of pancreatic [^18^F]FP-(+)-DTBZ uptake measurements in human pancreata. Two scans were carried out in five healthy control subjects and two patients with T1D. Repeat scans were performed less than 1 month after the initial scanning. The results showed that the mean variabilities on the measurements of BP_ND_ and the functional binding capacity were 9.4% and 16.6%, respectively. This result indicates that this method may not be sensitive for marginal changes in pancreatic mass since it was unlikely to have around 10% progress loss in this time window in the same individual. It is also necessary to include normalization of the PET signal to pancreatic volume in future studies.

These clinical trials also present an issue that the reductions (59–63%) in the tracer functional binding capacity in T1D patients relative to controls is lower than expected since it is known that in these T1D patients at least 90% of the beta cells are lost. Possible reasons for the increased tracer uptake in diabetes patients include higher than expected non-specific binding to the exocrine pancreas [18], radioactive metabolites as a confounding source of the signal, and pancreatic sites other than beta cells expressing VMAT2. While 90% of beta cells express VMAT2, approximately 40% of gamma cells also express VMAT2 [19]. The non-specific uptake of the tracer can be corrected by using an appropriate reference region, such as the spleen [20]. While there are some limitations and issues that need to be further investigated, these results suggest that longitudinal PET imaging of VMAT2 using [^18^F]FP-(+)-DTBZ has potential for non-invasively and quantitatively measuring BCM in patients with T1D.

#### 2.2.2. [^11^C]5-hydroxy-tryptophan ([^11^C]5-HTP)

[^11^C]5-HTP is the radiolabeled serotonin precursor and was first used as a PET tracer to assess the rate of serotonin biosynthesis for localization of neuroendocrine tumors, including insulinomas [21]. Although [^11^C]5-HTP is taken up by exocrine and endocrine tissues, it is selectively retained only in endocrine cells. The presence of serotonin synthesis machinery is required to retain [^11^C]5-HTP, which is present in the islet cells, but not in exocrine cells. Thus, the radiolabeled [^11^C]5-HTP is converted to a serotonin analog and is accumulated in the pancreatic islets of Langerhans, rather than exocrine pancreatic parenchyma. Therefore, it was hypothesized that [^11^C]5-HTP uptake could be used as an in vivo surrogate marker for observing pancreatic endocrine cells in humans [22].

In 2014, Eriksson et al. [22] reported a dynamic PET study using [^11^C]5-HTP for the quantification of human islet cells with 10 patients with T1D and 9 healthy volunteers (HVs). In this study, the pancreatic uptake of [^11^C]5-HTP in T1D subjects was significantly reduced (66%) when compared with that recorded in HVs, which was most apparent in the corpus and caudal regions of the pancreas where beta cells are normally the major constituent of the islets. It is suggested that complete loss of all beta cells would only partly reduce the endocrine signal because all residual pancreatic neuroendocrine tissue should also retain [^11^C]5-HTP, not only beta cells. In addition, when correcting for the pancreatic volume in each individual tracer accumulation per gram of pancreas (% ID/g), they reported that the pancreatic radioactivity in T1D patients was decreased 39% at 60 min after tracer administration compared to HVs, which was reduced 41% in the corpus and 47% in caudal parts of the pancreas and a lesser extent (34%) in the caput part. It is suggested that the volumetric contribution is likely the suitable parameter when assessing BCM and can be approximated to 62% (range 46–75%) by averaging previously reported values from nine studies using human pancreatic sections [23]. Thus, around 62% reduction of the [^11^C]5-HTP signal in this PET measurement should be considered as a complete loss of all beta cells. Furthermore, the serotonin machinery in other cell types including alpha cells needs to be further investigated. In this study, it was documented that the mean pancreatic blood flow in the T1D group was decreased by 20% compared with HVs, but there was no clear correlation between the uptake of [^11^C]5-HTP and the blood perfusion, which will be a subject for future study too. Overall, the results suggest that [^11^C]5-HTP is a potential PET tracer for the quantification of human islet beta cells [22].

In 2016 the same group reported their study using [^11^C]5-HTP as a PET tracer to monitor viable islet mass for 8 T1D patients who received an intraportal islet transplantation (IPX), and who underwent two PET examinations 8 months apart [24]. PET scans were 60 min and imaged over the abdominal region. The liver uptake of [^11^C]5-HTP was measured by delineating the liver on sequential co-registered CT images. It is found that the measurement of the whole-liver SUV was not able to correlate the tracer uptake in liver with metabolic function due to the dilution of the tissue and non-endocrine background signal. Thus, they inspected the livers of non-transplanted subjects with T1D as a control for the IPX patients. Based on the retrospective study of the uptake of [^11^C]5-HTP in the liver of non-transplanted patients with T1D, the average SUV plus 2 × SD (SUV = 2.09) was assumed to represent the maximal physiological background uptake in liver with T1D patients. In this method, all liver areas with a mean SUV > 2.09 in subjects with IPX were considered to predict hotspots, which could imply accumulation of the tracer in islets. The results indicated that the tracer uptake in hepatic hotspots had a correlation with metabolic assessments of islet function.

The second round of PET examinations and metabolic tests were accomplished 8 months after the first. The results showed that the change in hotspot SUV predicted loss of graft function in one subject, whereas the hotspot SUV was unchanged in subjects with stable graft function. The study demonstrated a correlation between the [^11^C]5-HTP uptake in liver and the transplanted islet cells function. 

This as well as the previous study suggested that [^11^C]5-HTP PET imaging has promise as a tool for non-invasive detection of both viable endogenous and transplanted islets [22,24].

#### 2.2.3. [Lys^40^(Ahx-DTPA-^111^In)NH_2_]exendin-4

Since glucagon-like peptide-1 receptor (GLP-1R) are highly expressed in pancreatic beta cells but not alpha, delta and PP cells, ligands of GLP-1R could be potential probe for pancreatic beta cell imaging [25]. Because endogenous ligand GLP-1 is degraded within minutes in vivo, many research efforts have been dedicated to improve its in vivo efficacy and its biological half-life. Exendin-4 is a more stable GLP-1R ligand with picomolar affinity that is a 39 amino acid peptide (Figure 1).

In 2010, Pattou et al. reported their SPECT study of GLP-1R using the [^111^In] labeled exendin-4 probe, [Lys^40^(Ahx-DTPA-^111^In)NH_2_]Exendin-4, for imaging the autologous transplanted islets in the left brachioradialis muscle of a patient, who underwent the resection of at least 80% of the pancreas for an insulinoma [26]. One year after transplantation, a whole-body planar scan was performed after intravenous administration of the radioligand [Lys^40^(Ahx-DTPA-^111^In)NH_2_]Exendin-4. The results demonstrated that focal accumulation of the radiolabeled GLP-1 probe was visible in the left forearm at the site of islet transplantation, showing its potential to evaluate islet survival in clinical transplantation.

In 2014, Brom et al. published their study for non-invasive quantification of the BCM by SPECT with an ^111^In-labelled exendin-4 probe [27]. This study was carried out with five T1D and five healthy controls, who were imaged with SPECT 4, 24, and 48 h after receiving the [^111^In]-labeled exendin-4. The tracer uptake was evaluated by quantitative analysis of the SPECT images. The results showed that the uptake of ^111^In-labelled exendin-4 was clearly observable in the pancreas. The pancreatic uptake of the tracer was clearly reduced in patients with TD1, although there was some overlap in pancreatic uptake between the groups and high individual variation. It was also revealed there was no significant difference in pancreatic uptake of the tracer at 4, 24 or 48 h after injection. The results indicated that SPECT imaging with this [^111^In]-labelled exendin-4 probe allowed non-invasive visualization of beta cells and measurement of BCM. In addition, the authors suggested that their SPECT procedure with the [^111^In]-labeled exendin-4 is a much simpler method, which can be carried out in every hospital equipped with a gamma camera, does not need a cyclotron and the labelled compound can easily be distributed ready to use for clinical studies. 

Clinical PET/SPECT imaging of BCM with various radiotracers have displayed some promising results. To track the dynamic BCM changes for transplanted islets, longitudinal measurement and quantification is necessary. The tracers applied for imaging has to be non-toxic and low radiation dose without damaging beta cells or other tissues. Radiodosimetry studies displayed that these probes had acceptable radiation induced damage to islets, including [^18^F]-FP-(+)-DTBZ [28] and [^68^Ga]Ga-DO3A-VS-Cys_40_-Exendin-4 [29,30]. For tracers used for tracking BCM after transplantation, there needs to be caution about the dosimetry to beta cells and surrounding tissues, as the volume of beta cells is extremely small, the local density of radiation may be high, leading to high dose to beta cells, especially for longitudinal studies with repeated administration of radiotracers.

To non-invasively image transplanted islets, alternative imaging modalities do not have ionization radiation involved have also been investigated, such as MRI, optical imaging of the anterior chamber of the eye (ACE) and ultrasound imaging.

## 3. Magnetic Resonance Imaging (MRI)

MRI has been tested to monitor transplanted pancreatic islets in clinic as well. Whereas nuclear imaging is characterized by high sensitivity and quantitative, MRI does not utilize ionizing radiation, has tomographic capabilities, delivers superior soft tissue contrast resolution in vivo, and has unlimited depth penetration. Although MRI imaging has a relative low sensitivity in detecting molecular probes (10^−3^−10^−5^ M), this drawback can be overcome by the application of contrast agents either direct labeling of the islet grafts or in vivo indirect labeling by targeting specific biomarkers on beta cells that amplify the signal. Superparamagnetic iron oxide nanoparticles have been extensively investigated as magnetic resonance reporters for tracking transplanted islets [31,32]. Their basic structure includes an iron oxide core covered with a dextran coat that can be functionalized with additional imaging, targeting, or even therapeutic moieties. By adding super paramagnetic iron oxide (SPIO) nanoparticles to the cell culture medium, islets cells can accumulate SPIO in their cytoplasm in a non-specific manner. The presence of iron oxides in cells or tissue is evidenced by a shortening of the T2 relaxation time of surrounding water protons [8,33].

In a 2008 study, SPIO nanoparticles used to label and monitor transplanted islets in 4 T1D patients using MRI. MRIs of the patients were performed prior to the transplantation and 5 days, 6 weeks and 6 months after transplantation or when a significant metabolic event occurred. After transplantation, labeled islets were on average 88% viable and all patients were able to stop treatment with externally delivered insulin. Patient 1, who received an islet transplant after a kidney transplant had diffuse hypointense images for baseline MRI. After the third post-transplant MRI the signal could not be found. Patient 1 also suffered from a decrease in islet graft function and after 15.5 months had to resume insulin injections, but there is no clear correlation between islet function and MRI signal. The other three patients, patient 2 who received a kidney and islet transplantation simultaneously, and patient 3 and 4 who only received an islet transplantation were observed with normal intensity on pre-transplant MRI, labeled islets were visualized in livers on post-transplantation MRI. The foremost limitation for this study was linked to the specific intraportal location of the islets which could be caused by presence of too much iron and background within the liver. This limitation contributes to the fact that no correlation was found the number of transplanted islets and number of spots in the liver. Observed islets were counted manually and varied between investigators, showing a need for an automated counting technique. Overall though the study showed the possibility that MRI could be used to safely images transplanted islets, and had islet-induced images for 6 months after the transplantation. The study also showed that further studied need to be completed and what areas need improvement for this to become a viable option for monitoring islet cell transplants [34].

In 2010, eight T1D patients received a SPIO nanoparticles labeled islet transplantation, with three patients receiving the islets after a kidney transplant and five just receiving islets. The islet cells were transplanted into portal vein and were monitored with MRI, one day post transplantation and 1, 4, and 24 weeks after. All patients had significant c-peptide and HbA1c were close to non-diabetic levels. Patients were able to decrease insulin dose levels 50–80% compared with pre-transplantation. Pancreatic islets represented by hypointense spots were apparent in the livers on MRI. There was a dramatic decrease in the relative number of spots one week after transplantation for all patients. The number of detected islets was low in relation to the number of labeled islet cells that were transplanted. The most likely reasons for this was that the islets being destroyed right after infusion, the islets are clustering together and being detected by the MRI as a single cell or the islets not being labeled properly. Islet quantification was only performed on images of four patients who had only received one transplant within the 24-week period. For the other two patients, transplanted islets were not quantified due to insufficient labelling time (only for 6 and 10 h). It is believed that at least 16 h is needed for proper islet labeling. In conclusion, even though improvement for the low correlation between the number of transplanted islets and number of detected islets by the MRI is needed for the data confirms that MRI is able to monitor the presence of transplanted pancreatic islets in the liver, over a period of months [35].

In 2015, three T1D and one non-diabetic subjects received SPIO nanoparticles labeled islets, monitored with MRI and metabolically 1-, 3- and 7-days post transplantation. Patients 1 and 3 were observed with notable midterm graft loss. Patient 1 was insulin free for 6 months, following a second islet cell transplant. Patient 3 also received a second islet transplant at 6 months post first transplant, but never became insulin free. Patient 2 experienced complete disappearance of all hypointense signals at 28 days after a peak of alanine aminotransferase (ALT) and aspartate aminotransferase (AST) at day 21. Four main points came from this study: (1) SPIO nanoparticle-labeled islets are safe for both in vitro and in patients; (2) in the short-term region of interest (ROI) number parallels the number of successfully engrafted islets; (3) 20–30% of islet numbers are lost in the first few days after an allotransplant; (4) it is difficult to assess the appropriateness of using SPIO nanoparticles labeling for mid-to long-term islet survival monitoring. There are some limitations with the results of this study due to such a small sample size. This was due to the production of Endorem^®^ (Guerbet, Sulzbach, Germany), SPIO nanoparticles used in this study stopping during the trial. Because of this more studies will be need to be done using with larger patient groups and newer contrast agents. Overall the results suggest that MRI monitoring of islet transplantation at early times could represent a meaningful readout for helping a predicting transplant failure or success [36].

Theranostic is a combination of diagnostic and therapy. The potential for theranostic has already been explored in diabetes including islet transplantation [37,38] and endogenous beta cell drug delivery [39]. Recently, one study demonstrated a theranostic approach aimed at protection of islet grafts in non-human primates by silencing a gene (caspase 3), which is a major player for cell apoptosis. The small interfering RNA (siRNA) targeting caspase-3 were conjugated to magnetic nanoparticles named MN-siCas-3. Baboon islets were labeled with MN-siCas-3 and transplanted to diabetic subjects. A dramatic reduction in insulin requirements was observed in baboons transplanted even with a marginal number of labeled islets compared to controls. These nanoparticles not only served as a vehicle for siRNA delivery but also acted as imaging probe for MRI monitoring in vivo. This pre-clinical study provided a novel strategy donor islets protection and follow up [40].

## 4. Optical Imaging and Ultrasound Imaging

Recently, a clinical trial has initiated with a cohort of 10 persons to test the pancreatic islet transplantation into the anterior chamber of the eye (ACE) (NCT02846571). This ACE technology provided a platform transplanting pancreatic islets into the ACE where they later on can be imaged non-invasively with optical imaging tools over a long time [41]. Thus ACE technology contains two parts, transplanting of islets into the ACE and imaging of islets engrafted in the ACE. The source of islets is mostly allogenetic. After the islets are genetically labeled with biomarkers or biosensors, the islets are transplanted into ACE for imaging or glycocontrol. The transparent cornea provides a window for the observation of intraocular islets under physiological conditions, using otherwise in vitro imaging tools such as confocal/multiphoton microscopy. However, the ACE imaging technology was limited by the depth of view, and could not reach the full resolution capacity of confocal/multiphoton microscopy due to the movements of islets caused by heartbeat, respiration, pupil movement. Anesthesia can significantly reduce these movements, however it may produce side effects. Thus, the ACE technology does not apply to awake animals. 

The ACE is not only a convenient optical imaging site, it also offers an immune privileged niche, oxygen-rich milieu and metabolic stress reducing environment where intraocular islets survive better and become functionally stronger. In both rodents [42] and non-human primates [43], islets underwent optimal engraftment, rich vascularization and dense innervation, preserve organotypic features and live with satisfactory viability and functionality. The great animal results enable the clinical trial of ACE technology in humans to further demonstrate its clinical value. This novel technology will substantially contribute to the clinical imaging of BCM after islet transplantation to ACE.

Ultrasound imaging has been used for islet transplantation. It is reported on a clinical case, who had chronic pancreatitis and received total pancreaectomy with autologous islet transplantation. Intraoperative ultrasound examination was undertaken to detect transplanted islets, which were revealed as hyperechoic clusters that flow from the tip of the catheters into the portal vein [44]. Ultrasound imaging could also be used for monitoring focal liver fatty infiltration that happened post islet transplantation [45]. Contrast-enhanced ultrasound has also been used for detecting inslulitis in pre-clinical models of T1D, which shows accumulation of nanobubbles specifically within pancreatic islets, correlating with insulitis. Thus, nanobubble ultrasound imaging provides a marker for disease progression for T1D [46].

## 5. Conclusions and Perspectives

Pancreatic islet transplantation has the potential to be a cure for T1D; however, without the ability to image the cells post-transplantation, the studies are inadequate. The capability of researchers to be able to in real-time monitor clinically transplanted islet cells could lead to a greater understanding of the epidemiology of diabetes, along with the potential for finding a cure with transplanted islets. The imaging techniques and tracer discussed in this article all have their strengths and weaknesses and will need further studies to be undertaken to observe their full potential for being the primary imaging model for islet cells.

For the ex vivo labeling approach, the prelabeled islet cells with [^18^F]FDG has allowed the PET imaging to track and quantify BCM for a real-time monitoring of the intraportal islet transplantation, although there were some limitations regarding partial volume effect and potential signal spill-over [13]. On the other hand, although significant progress has been made for the in vivo targeting approach, only three radio-ligands have been advanced into clinical trials of human beta cell imaging. There were also concerns regarding controversial non-specific signals reported in previous studies with results using the [^18^F]FP-(+)-DTBZ as tracers [16]. The studies with [^11^C]5-HTP had showed that the tracer could be used to noninvasively quantify islet cells [13,24]. However, there were some limitations as well, it was thought that [^11^C]5-HTP could have issues with the interpretation of the lower accumulation of radiation signals from ROIs because of a variety of factors leading to the results, including decrease in pancreatic blood flow in T1D patients. The SPECT tracer, [Lys^40^(Ahx-DTPA-^111^In)NH_2_]exendin-4, displayed some promising results to be used as a non-invasive imaging model for pancreatic BCM and transplanted islet cells in the muscle [26,27]. However, more studies need to confirm this method and to study the possible application to imaging the islet transplantation in the liver. Since these clinical trials have based on different medical design and a small number of subjects, it is hard to compare different probes. Further studies will be necessary to demonstrate the potential of the radiotracers outlined above as the markers for human beta cells.

MRI monitoring transplanted SPIO labeled islets have been performed in patients [34,35]. However, despite the overall safety of transplanted islets labeled with iron oxide nanoparticles, image interpretation and quantification of the number of transplanted islets remain challenging. Signal voids in magnetic resonance (MR) images produced by iron labeled islets/islet clusters were difficult to distinguish from other low MR signals produced by tissue including intestine and blood vessel structures or artifacts. In addition, negative contrast has contributed to the poor MRI T2* quantification of the number of infused islets, as noted in prior MRI clinical studies [34,35].

Recently, a simultaneous PET/MRI protocol to comprehensively quantify in vivo changes in BCM by targeting GLP-1R and voltage-dependent calcium channels (VDCC) has been developed. Differences in the spatial distribution of [^64^Cu] exendin-4 and Mn were monitored over time in native and spontaneous pancreatic neuroendocrine tumor models. Simultaneous PET/MR imaging of the pancreas enabled the comprehensive in vivo quantification of BCM using radiolabeled exendin-4 and Mn. The results showed that only late time-point measurements reflect the Mn uptake in the beta cells, while early time points detect non-specific accumulation of Mn in the exocrine pancreas. Such a dual imaging approach enables the correlation of the comprehensive imaging information at high spatial and temporal resolution [47].

In addition, a newer imaging modality that was introduced in 2005 shows promise for the tracking of cell transplants. Magnetic particle imaging (MPI) is an imaging method used to for detecting SPIO nanoparticles with advantages including: high specificity and sensitivity, the absence of the background signal, linear quantitative ability, and high potential for clinic translation. This new imaging technology has already been utilized for stem cell tracking [48,49] and islet transplantation [50], and other indications. MPI’s great specificity results from its high image contrast, since magnetic particles serve as the only source for signal and are thus the only visualized element [51]. MPI’s high sensitivity derives from the direct detection of the electronic magnetization of SPIO nanoparticles, which is 10^8^ times larger than the nuclear magnetization of protons seen in MRI [52,53]. This translates to an MPI sensitivity in the hundreds of cells with current hardware and available magnetic nanoparticles. MPI’s linear quantitation arises from the linear signal change with nanoparticle concentration, which occurs independent of tissue depth. We anticipate that MPI will be used in addition to MRI (i.e., MPI does not replace MRI, it simply augments MRI as an extra layer of information, like PET/MRI) and is very promising for clinical applications in the future [54,55]. 

In addition to propagating further analysis of medical imaging involving transplanted islets and cell tracking, advances in artificial intelligence (AI) and machine learning, specifically deep learning, can provide newfound insight and rapid, high throughput analysis of medical imaging of transplanted islets. The concept of deep learning, with the use of advanced algorithms such as convolutional neural networks (CNN), has previously provided a great deal of insight in molecular imaging domains such as MRI and CT [56,57,58,59]. These algorithms are beginning to be applied to imaging modalities such as PET and MPI, with advances being made not only in segmentation and analysis of regions of interest (ROI) within a particular image, but also classification of target lesions/transplants and early disease prediction and diagnosis [60,61,62,63]. This is important in permitting the use of these algorithms and its applicability to longitudinal studies and analysis thereof, a rather important facet of monitoring of transplanted islet grafts [64]. This computationally advanced tool can have a synergistic effect in increasing the current approach to monitoring and analysis of transplanted human islets and provide new methods of insight into cellular tracking through molecular imaging modalities.
